# An Interpretable Approach with Explainable AI for Heart Stroke Prediction

**DOI:** 10.3390/diagnostics14020128

**Published:** 2024-01-05

**Authors:** Parvathaneni Naga Srinivasu, Uddagiri Sirisha, Kotte Sandeep, S. Phani Praveen, Lakshmana Phaneendra Maguluri, Thulasi Bikku

**Affiliations:** 1 Department of Teleinformatics Engineering, Federal University of Ceará, Fortaleza 60455-970, Brazil; 2 Department of Computer Science and Engineering, Prasad V Potluri Siddhartha Institute of Technology, Vijayawada 520007, India; sirisha.uddagiri@gmail.com (U.S.); sppraveen@pvpsiddhartha.ac.in (S.P.P.); 3 Department of Information Technology, Dhanekula Institute of Engineering & Technology, Vijayawada 521139, India; kottesandeep@gmail.com; 4 Department of Computer Science and Engineering, Koneru Lakshmaiah Education Foundation, Vaddeswaram, Guntur 522302, India; phanendra51@kluniversity.in; 5 Computer Science and Engineering, Amrita School of Computing Amaravati, Amrita Vishwa Vidyapeetham, Amaravati 522503, India; thulasi.bikku@gmail.com

**Keywords:** Artificial Neural Network, deep learning, data leakage, sampling, feature selection, explainable AI, LIME tabular

## Abstract

Heart strokes are a significant global health concern, profoundly affecting the wellbeing of the population. Many research endeavors have focused on developing predictive models for heart strokes using ML and DL techniques. Nevertheless, prior studies have often failed to bridge the gap between complex ML models and their interpretability in clinical contexts, leaving healthcare professionals hesitant to embrace them for critical decision-making. This research introduces a meticulously designed, effective, and easily interpretable approach for heart stroke prediction, empowered by explainable AI techniques. Our contributions include a meticulously designed model, incorporating pivotal techniques such as resampling, data leakage prevention, feature selection, and emphasizing the model’s comprehensibility for healthcare practitioners. This multifaceted approach holds the potential to significantly impact the field of healthcare by offering a reliable and understandable tool for heart stroke prediction. In our research, we harnessed the potential of the Stroke Prediction Dataset, a valuable resource containing 11 distinct attributes. Applying these techniques, including model interpretability measures such as permutation importance and explainability methods like LIME, has achieved impressive results. While permutation importance provides insights into feature importance globally, LIME complements this by offering local and instance-specific explanations. Together, they contribute to a comprehensive understanding of the Artificial Neural Network (ANN) model. The combination of these techniques not only aids in understanding the features that drive overall model performance but also helps in interpreting and validating individual predictions. The ANN model has achieved an outstanding accuracy rate of 95%.

## 1. Introduction

A heart stroke, also called a cerebrovascular accident (CVA) or brain stroke, is a serious medical condition in which blood flow to the brain suddenly ceases, damaging the cerebral cells. Numbering among the leading causes of death and disability in the world, strokes [1] are a major health problem. Every year, there are roughly 13.7 million new instances of stroke, as reported by the World Health Organization (WHO). It is estimated that nearly 5 million people die from strokes every year. Ischemic stroke is responsible for approximately 87% of all instances of stroke. The amount of blood that can flow to the brain is reduced by clots or plaques that block blood vessels. Strokes caused by hemorrhage are caused by a ruptured blood vessel in the brain, causing internal or external bleeding. The major risk factor for stroke that may be modified is hypertension, also known as high blood pressure. A variety of factors, such as smoking, diabetes, obesity, excessive alcohol consumption, high cholesterol, low activity levels, and insufficient physical activity also increase the risk of heart disease.

Strokes [2,3] can occur in people of any age, although the likelihood of having one rises with advancing years. Stroke is the greatest cause of mortality among women, even though males have a slightly larger risk of experiencing one than do women. It is common for stroke patients to experience sudden numbness or weakness on one side of their bodies. Confusion, difficulty speaking or understanding speech, severe headaches, and difficulty walking are some of the additional symptoms that may be encountered. Stroke is one of the primary causes of impairment that lasts a long time, and survivors may face difficulties in their physical, cognitive, and emotional functioning [4,5]. The mortality rate associated with stroke can vary significantly based on many factors, including the type of stroke, the amount of time that passes before treatment begins, and the pre-existing diseases of the patient. Modifications to one’s way of life, such as consuming a nutritious diet, engaging in regular physical activity, and controlling risk factors, can help lower one’s stroke risk. To manage risk factors, patients may be offered blood-thinning and antihypertensive medications.

### 1.1. Problem Statement

Heart stroke is often associated with fluctuations in blood pressure and cholesterol levels within the body. Some proactive strategies, like adopting a heart-healthy diet and embracing a physically active lifestyle, can effectively mitigate the risk factors contributing to heart strokes. Detecting the early signs of heart-related issues can be facilitated through regular medical check-ups and specialized laboratory tests designed to assess cardiovascular health. For individuals with specific risk factors, including hypertension or a family history of heart disease, timely intervention is crucial. The untreated consequences of heart strokes can have far-reaching impacts, affecting not only the individuals affected but also placing a strain on their families and national healthcare resources. Hence, early identification and implementing appropriate preventive measures are pivotal in safeguarding the wellbeing of individuals at risk of heart stroke [6,7,8]. Integrating intelligent systems that consider both symptoms and diagnostic tests can significantly aid in the early diagnosis and prevention of heart-related conditions, potentially saving lives and reducing the burden on healthcare systems.

### 1.2. AI Challenges in the Field of Heart Strokes

Artificial Intelligence (AI) has the potential to analyze a range of factors, including an individual’s medical history, risk factors, and results from diagnostic tests. This analysis aims to evaluate an individual’s susceptibility to experiencing a heart stroke. AI algorithms can process extensive datasets, encompassing vital signs and medical records, to pinpoint individuals at risk of suffering from a heart stroke. Developing an intelligent machine learning-based diagnostic approach is also feasible for predicting heart strokes. Regarding heart stroke prediction, comparable challenges can surface, with the selection of appropriate AI algorithms and the quality of input data emerging as pivotal factors in attaining precise predictions. Healthcare professionals must comprehend the rationale behind an AI model’s predictions, as this comprehension informs their decision-making process in patient care. In this context, healthcare practitioners must have confidence in and grasp the insights provided by AI-driven predictions to deliver the highest quality care to their patients. AI holds significant potential in heart stroke prediction and diagnosis; however, it must confront parallel challenges to ensure precision and interpretability in its application by healthcare professionals.

### 1.3. Research Drive

Several studies have been conducted using the Stroke Prediction Dataset in recent years, and the results have been positive. Train–test splits are relatively straightforward to implement when the dataset is characterized by pronounced variability. Nevertheless, obtaining consistently high accuracy remains a formidable challenge when utilizing the cross-validation (CV) approach. Additionally, the dataset exhibits an imbalanced class distribution, necessitating effective class-balancing techniques to mitigate potential overfitting or underfitting. Lastly, despite substantial ongoing research, the challenge of interpretability persists in existing ML detection and progression prediction.

### 1.4. Objectives, Contribution, and the Structure of the Paper

Heart stroke is a pervasive and serious health concern globally, impacting the overall well-being of the population. Numerous research efforts have been made to develop effective predictive models for heart strokes using machine learning (ML) and deep learning (DL) techniques. The proposed approaches aim to improve accuracy by incorporating resampling techniques, preventing data leakage, and implementing feature selection. However, these studies have often fallen short in influencing clinical practice at the expense of their interpretability in clinical settings. Consequently, physicians struggle to comprehend these models and hesitate to rely on them for clinical decision-making. This research introduces a meticulously designed, effective, and easily interpretable approach for heart stroke prediction, leveraging explainable AI techniques.

The most important contributions made by this research can be summarized as follows:

- The proposed model introduces a meticulously designed, effective, and easily interpretable approach for heart stroke prediction, leveraging explainable AI techniques;
- Model quality and effectiveness can be enhanced by using several techniques in ML and DL. The proposed approach has incorporated techniques such as resampling, data leakage prevention, and feature selection, which are significant;
- To enhance the model’s reliability and balance accuracy and interpretability, we provided insight into the model’s internal workings. The model is, therefore, easier for healthcare professionals to understand and apply.

An outline of the sections that follow in chronological order is provided below:

- The second section provides an overview of the most recent research in the topic;
- Our suggested methodology is broken down in Section 3, including explanations of datasets and methods;
- The performance of the model is presented in Section 4;
- In this report’s fifth and last section, we summarize the most important findings from our investigation and discuss new potential lines of inquiry for further study.

## 2. Literature Review

Scientists have been exploring diverse ML methodologies for early disease prediction. Numerous ML algorithms, including hybrid methods, have been devised to enhance the performance of predictive models. The Stroke Prediction Dataset has been a common choice among researchers in this domain, and this section provides an overview of relevant studies conducted in this area.

In a study documented in [9], the Cardiovascular Health Study (CHS) dataset was utilized, employing five distinct ML techniques. Their research revealed that the most favorable outcomes were obtained by integrating decision trees with principal component analysis, artificial neural networks, and support vector machines. It is worth noting that the CHS dataset limited the number of input parameters. Another approach, as detailed in [10], involved the application of the detecting risk factor of stroke disease (DRFS) technique to extract information about stroke symptoms from social media posts. This method employed natural language processing (NLP) to extract text from comments, which increased the model’s processing time. In the study discussed in [11], the authors introduced a modified version of the random forest algorithm for stroke prediction, demonstrating its significant performance improvement compared to previous methods. However, this study had limitations, including a focus on a specific subset of strokes and a potential lack of adaptability to future advancements in the field. Three machine learning models were observed to have 74% to 75% accuracy in [12], decision trees, random forests, and multi-layer perceptrons. The study slightly favored the multi-layer perceptron, although it solely used accuracy as an evaluation criterion, which may not always suffice. In [13], stroke prediction was explored using decision trees, naïve Bayes, and SVM, with a maximum accuracy of 60%. In contrast, [14] employed three data mining classification algorithms—C4.5, Jrip, and multi-layer perceptrons (MLP)—achieving a notable accuracy of 95%. However, this high precision was achieved by combining intricate algorithms, consequently extending the training and prediction times.

Stroke prediction strategies employing naive Bayes, decision trees, and neural networks were examined in [15]. The decision tree algorithm exhibited the highest accuracy at 75%, although the model’s practical utility was questioned due to insights from the confusion matrix. A distinctive approach in [16] proposed an automatic feature selection method for stroke prediction using the CHS dataset. This algorithm conservatively selected robust features, but when combined with the support vector machine, it led to an overwhelming number of vectors, diminishing the model’s effectiveness. Finally, [17,18,19] employed the backpropagation algorithm with artificial neural networks (ANN) to precisely predict thromboembolic strokes. However, as the complexity of neural networks increases with more neurons, training them becomes more challenging and resource-intensive.

## 3. Proposed Methodology

This section provides a succinct overview of the experimental data, their interpretation, and possible experimental inferences.

### 3.1. Proposed Approach

**Figure 1:**
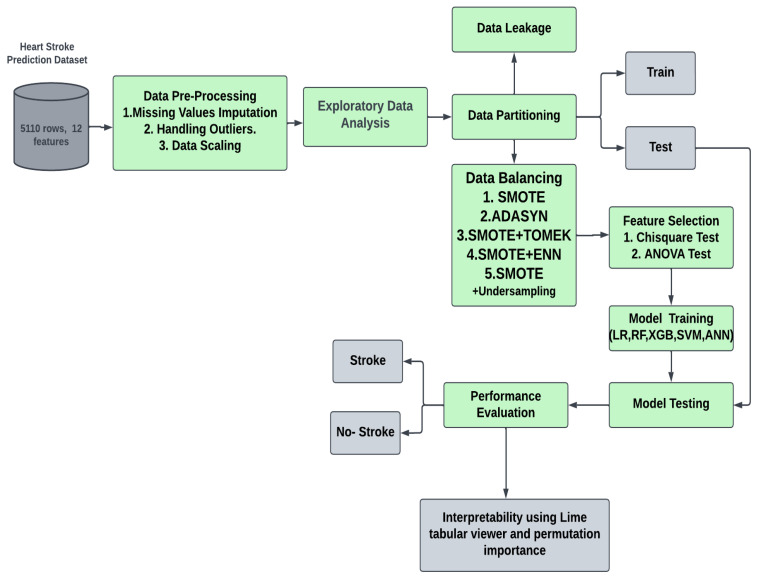
The overall workflow of the proposed stroke prediction model.

Figure 1 depicts the whole workflow of the proposed technique in its entirety. After obtaining the data from Kaggle (https://www.kaggle.com/datasets/fedesoriano/stroke-prediction-dataset, [20] accessed on 30 September 2023), the information was cleaned and preprocessed in several different ways, including the treatment of missing values and the correction of class imbalances.

Model quality and effectiveness were enhanced through various techniques used in ML and data analysis. Among these, resampling techniques, data leakage prevention, and feature selection are significant. Resampling methods, which include oversampling, undersampling, and SMOTE, address the challenge of class imbalance in datasets. This is crucial when one class is notably underrepresented, ensuring balanced model training. Data leakage prevention is another essential step to safeguard against the inadvertent mixing of information between training, validation, and test datasets, ultimately guarding against overfitting. Additionally, feature selection techniques help streamline the modeling process by identifying and retaining the most relevant attributes, enhancing model efficiency and interpretability while mitigating the risk of overfitting. Collectively, these techniques contribute to robust, reliable, and more practical machine learning models suitable for real-world applications [21,22].

### 3.2. Feature Analysis

This study was sourced from Kaggle’s Stroke Prediction Dataset. There are 5110 rows in all, along with 12 columns. The following categories are represented in the columns: ‘Id’, ‘Gender’, ‘Age’, ‘Bmi’, ‘Hypertension’, ‘Heart_Disease’, ‘Ever_Married’, ‘Work_Type’, ‘Avg_Glucose_Level’, ‘Residence_Type’, ‘Smoking_Status’, and ‘Stroke’. The variable of interest is called ‘stroke’, it takes on a binary form, with a value of ‘0’ indicating that there is no risk of stroke and a value of ‘1’ indicating a risk of stroke. The dataset has a large class imbalance, with class ‘0’ having 4861 instances and class ‘1’ only having 249 instances. This disparity is noteworthy. This class imbalance has been addressed by applying data pre-processing to improve the accuracy of predictive modeling.

Table 1 provides a clear overview of each attribute in the dataset, including its data type and a brief description of its meaning and possible values. Table 2 provides statistical information for several attributes in the dataset. For the “ID” attribute, there are 5110 data points, with a mean value of approximately 36,518. Based on the same data points, the age attribute has an average age of around 43.23 years, with a standard deviation of approximately 22.61. The “HYPERTENSION” attribute, which is binary (0 or 1), has a mean value of approximately 0.097, indicating that about 9.7% of the data points have hypertension.

Similarly, the “HEART_DISEASE” attribute, also binary, has a mean value of around 0.054, suggesting that approximately 5.4% of the data points indicate the presence of heart disease. The “AVG_GLUCOSE_LEVEL” attribute has a mean value of approximately 106.15, with values ranging from 55.12 to 271.74. However, the “BMI” attribute has 4909 data points (indicating missing values) with an average BMI of approximately 28.89 and a standard deviation of approximately 7.85. Finally, the “STROKE” attribute, which is binary, has a mean value of about 0.049, indicating that approximately 4.9% of the data points represent instances of stroke.

### 3.3. Data Insights

**Figure 2:**
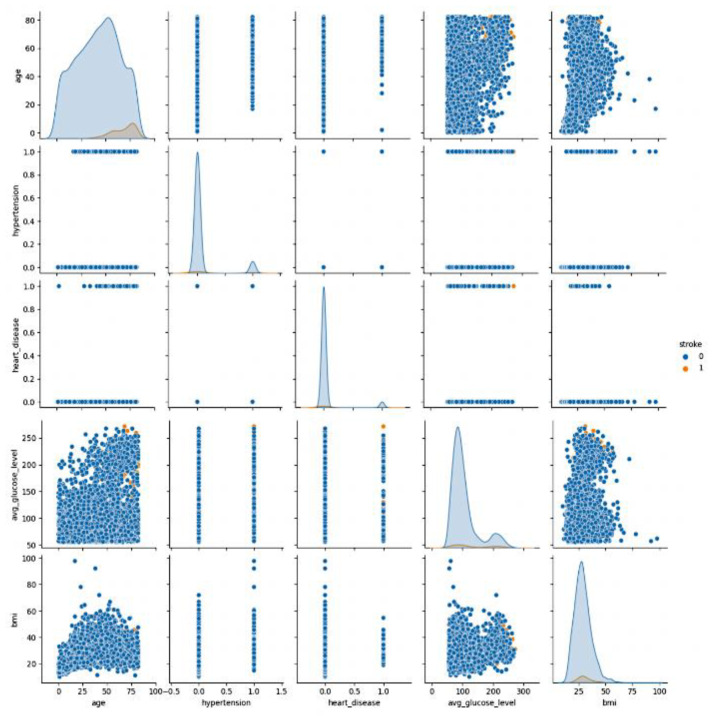
Graph depicting attributes in the Stroke Prediction dataset (outcome 0: no stroke, outcome 1: stroke).

Figure 2 displays pair plots for each feature, illustrating their relationships with the other features, including themselves. These plots serve as a valuable tool for identifying feature relationships. When data points are scattered across the plot, it indicates a lack of a strong relationship between the features. As a result, a line connecting the points suggests a linear relationship. In this context, when examining the pair plot, two features stand out as having a particularly strong positive correlation. These features exhibit a notable tendency to move together linearly, signifying their interdependence or association in the dataset.

**Figure 3:**
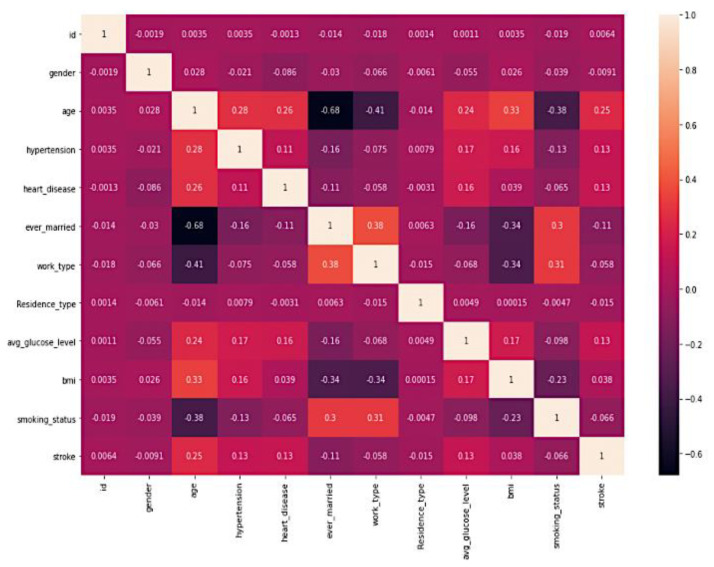
Heatmap of all attributes in the Stroke Prediction Dataset.

The Pearson correlation heatmap [23], which investigates the linear relationship between all of the features, is depicted in Figure 3. The Pearson correlation coefficient, which ranges from −1 to +1 and is used to quantify the link between pairs of features, is used to compute this correlation. This coefficient can take on a value between 0 and 1. A coefficient value that is closer to 0 shows that there is either no connection or a lesser correlation. In contrast, values that are closer to +1 or −1 indicate that there is either a stronger positive or negative correlation.

### 3.4. Data Pre-Processing

Data pre-processing is crucial for enhancing data quality, reducing noise, and ensuring accuracy in machine learning models. As part of this process, features are selected, cleaning is performed, missing values are handled, scaling is carried out, categorical variables are encoded, and missing values are handled. Effective data pre-processing lays the foundation for robust and reliable model training and evaluation.

#### 3.4.1. Missing Data Handling

**Figure 4:**
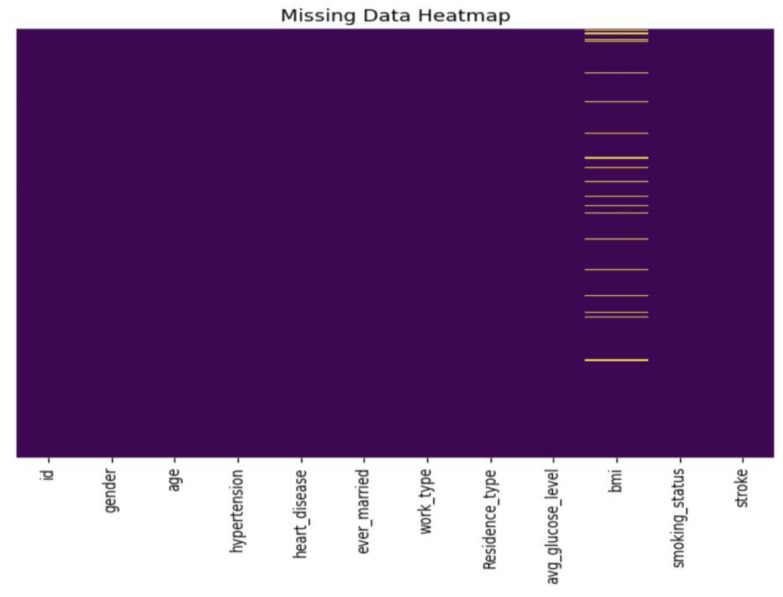
Distribution of missing data through a heatmap.

Among the 5110 total records, 201 missing BMI values were shown in Figure 4, which were imputed using Scikit-learn’s Simple Imputer with the median as a replacement. The ‘id’ column, deemed inconsequential, was removed. Additionally, an outlier was identified in the ‘gender’ attribute with the label ‘Other’ and was subsequently excluded.

In summary, our data pre-processing comprised:

- Detecting and addressing missing values;
- Eliminating the ‘id’ column;
- Handling outliers.

These measures are critical for ensuring data integrity and optimizing machine learning model performance.

#### 3.4.2. Handling Imbalanced Data

We used a method known as SMOTE Tomek [24,25], which combines the SMOTE (synthetic minority oversampling technique) and Tomek algorithms, to generate a balanced dataset in this study. Various methods may be used to accomplish this goal; however, we used one of these methods in this particular investigation. Tomek is an undersampling method, whereas SMOTE is a methodology that generates synthetic samples from members of minority classes to address class imbalance. Initially, SMOTE was used to achieve a more even distribution of classes by introducing new synthetic instances from the minority class. This was accomplished by creating new synthetic instances using the minority class. In addition, we used the Tomek link to exclude samples that were positioned close to the boundary that divides the two classes, which finally improved the separation between these classes [26]. This experiment only modified the training dataset, while the test dataset was left unchanged. As shown in Table 3, SMOTE is used both before and after training to compare the data distribution within each class. This comparison takes place before and after applying the method.

#### 3.4.3. Data Leakage

Data leakage [27] arises when external information is incorporated into the model-building process using data from outside the training dataset. Unfortunately, it is a frequently overlooked issue. Addressing data leakage is imperative for creating robust models, as relying on it often results in overly optimistic but practically unusable models that cannot be deployed in production environments. When data leakage is not properly managed, model performance deteriorates when deployed online. Although it may appear trivial, understanding this concept can be challenging. Dataset transformations include filling missing values with means, medians, modes, standardizations, normalizations, etc. However, this can result in data leakage if the processes are executed without considering the yet-to-be-seen test data. The training data should be split before any transformations to prevent data leakage. These transformations should be applied to training and test datasets based on the training data. Additionally, using k-fold cross-validation is encouraged to mitigate data leakage risks.

**Figure 5:**
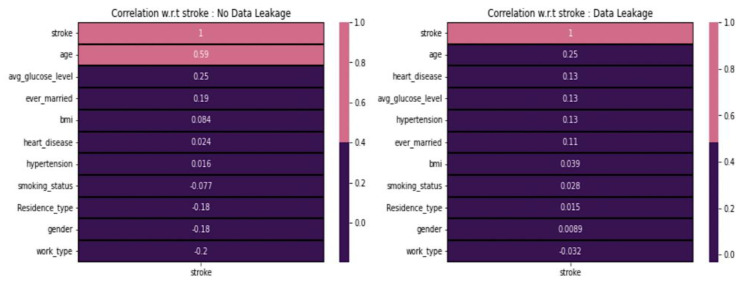
Data leakage concerning various features.

Figure 5 shows a noticeable distinction when comparing values between scenarios with and without data leakage. We observe that age has a strong correlation with stroke, whereas the ever married and average glucose level categories show some kind of correlation. In contrast, gender, residence-type, and work-type are negatively correlated with strokes. However, none of the features demonstrate an extreme positive or negative correlation with stroke in data leakage cases. Instead, the categories age, heart-disease, average glucose level, hypertension, and ever-married exhibit some form of positive correlation. All the features generally display correlation values close to zero, indicating a neutral correlation with stroke.

#### 3.4.4. Feature Selection

Feature selection for categorical features [28,29,30] involves choosing the most relevant and informative categorical variables to include in a predictive model while excluding less relevant ones. Mutual information measures the dependency between two variables and can be used to evaluate the relevance of categorical features. Features with higher mutual information with the target variable are more informative.

**Figure 6:**
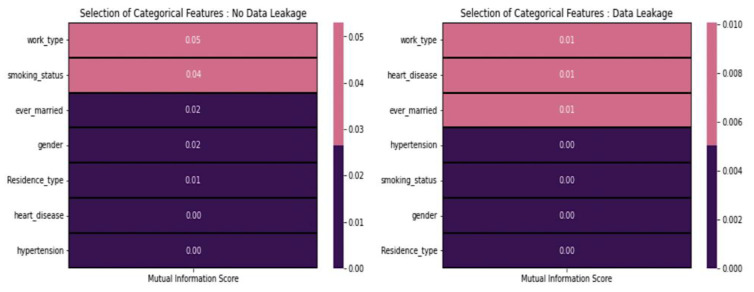
Mutual information score concerning features.

From Figure 6, the mutual information score between stroke and categorical features indicates consistently low values, regardless of the presence or absence of data leakage. Based on these scores, it is advisable not to include any of these features in the modeling process. Each categorical feature can be analyzed by using the chi-square test of independence [31]. Features with significant chi-square values are considered relevant.

**Figure 7:**
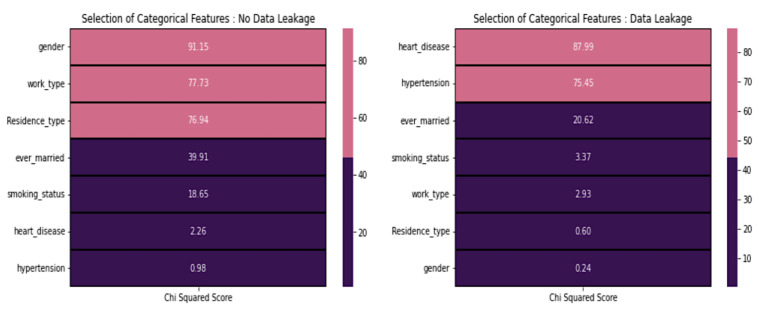
Chi-square test score concerning various features.

From Figure 7, in the case of no data leakage, it is recommended to exclude features with low scores, specifically those scoring below 20. Consequently, we omitted the following features: smoking_status, heart_disease, and hypertension. However, it is worth noting that this contradicts the domain-specific information. On the other hand, it is advisable to include heart disease and hypertension in the modeling process when dealing with data leakage, as they exhibit higher chi-squared scores than other features with lower scores.

#### 3.4.5. Feature Selection for Numerical Features

ANOVA, or analysis of variance, is a statistical test used to analyze the variation between two or more groups or treatments to determine whether they are significantly different from each other. ANOVA is often used in hypothesis testing to assess the equality of means among multiple groups.

**Figure 8:**
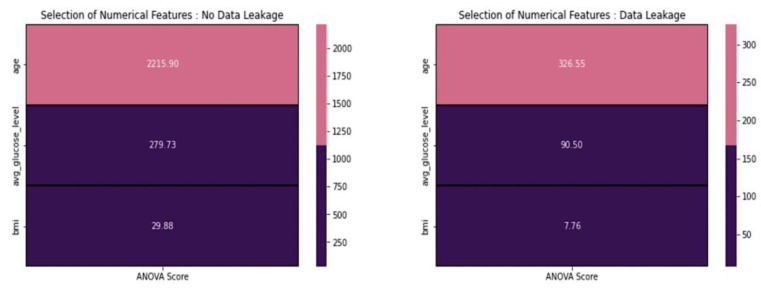
ANOVA score concerning various features.

From Figure 8, based on the ANOVA scores provided above, we excluded features with scores below 20. Consequently, regardless of the presence or absence of data leakage, we chose not to include BMI in our modeling. Based on the statistical tests above, we removed features from the datasets to prepare them for data scaling. In this process, we prioritized the statistical results over domain-specific information.

#### 3.4.6. Data Scaling

Because they treat feature values as numerical inputs without interpreting their significance, machine learning models do not know how to interpret them. Therefore, it becomes essential to scale the data appropriately. There are two main options for data scaling:

- Normalization: features with non-normal (Gaussian) distributions can benefit from this method;
- Standardization: standardization is used for features that exhibit a normal distribution but have values that are significantly larger or smaller in range compared to other features.

In addition to tree-based algorithms like random forests and XGBoost (XGB) classifiers, we normalized the dataset using min-max. Normalization was specifically applied to SVM, logistic regression, and ANN algorithms, as these models benefit from it.

### 3.5. Model Building

#### 3.5.1. Random Forest

Regarding the generation of base learners, the boosting technique known as random forest uses parallel ensemble methods. Under this strategy, each base learner model is allowed to autonomously work on a data sample, producing individual predictions. In the end, the conclusive prediction is arrived at through a voting classifier that considers the forecasts provided by all of the base learners. RF will build many decision trees and then aggregate the results of those trees to obtain a more accurate and reliable prediction. The decision trees serve as the foundational learner models for the random forest algorithmic architecture. The major objective of parallel approaches such as RF is to take advantage of the independence possessed by base learners to drastically cut down on errors through averaging. The Gini impurity for dataset *D* can be expressed in Equation (1).

(1) $$Gini\left(D\right)=1-\sum_{i=1}^{c}p_{i}^{2}$$

From the above equation, D represents the stroke prediction dataset, c is the number of classes, and p denotes the probability of class within the dataset D.

#### 3.5.2. XGBoost

XGBoost iteratively builds new models and then incorporates those models into an ensemble model. Initially, it works backwards from a previously constructed model to determine the residual errors for each observation. It builds a new model to anticipate those residuals by using the errors that have been made in the past. Then, the forecasts generated by the newly developed model are added to the ensemble. Because it can strike a balance between bias and variance properly, XGBoost stands out compared to other gradient-boosting algorithms and is recommended. For a binary classification problem, where the labels are either 0 or 1, the most common objective function used in XGBoost is the binary logistic loss. The objective function for XGBoost is expressed in Equation (2).

(2) $$F\left(x\right)=\sum_{j=1}^{m}{[(y}_{j})\log\left(p_{j}\right)+(1-y_{j}){(y}_{j})\log\left({1-p}_{j}\right)]+\sum_{k}^{K}\Omega \left(F_{k}\right)\;\;$$

In the above equation, the notation m is the number of training samples, the notation $y_{j}$ is the true label of the $j^{th}$ sample (0 or 1), $p_{j}$ is the predicted probability of the $j^{th}$ sample belonging to class 1, *K* is the number of leaves in the tree, $\Omega \left(F_{k}\right)$ is the regularization term that penalizes complex models, where $\left(F_{k}\right)$ represents the output score of the $k^{th}$ tree.

#### 3.5.3. Logistic Regression

The logistic regression (LR) transformation procedure within the linear regression framework offers a probabilistic interpretation for binary data. It performs the function of a classification algorithm by establishing a connection between various characteristics and the probability of a particular outcome. This classification approach utilizes the logit function, where the term “Logistic” comes from. LR is quite helpful in medical diagnostics, particularly when considering particular symptoms and qualities. Like other types of regression analysis, the likelihood ratio (LR) analysis belongs to the field of predictive analysis; specifically, it computes the probability that a result will occur. It exemplifies a particular implementation of linear regression developed for a categorical target variable. Logical regression uses the logit function, which reduces the influence of outliers. Logical regression is a type of multiple regression. The objective function for logistic regression is typically the log–loss (or cross–entropy) function in Equation (3).

(3) $$J(\theta )=-1/n\sum_{j=1}^{n}\left[y^{\left(j\right)}\log\left(h_{\theta }\left(x^{\left(j\right)}\right)\right)+\left(1-y^{\left(j\right)}\right)+log\left(h_{\theta }\left(x^{\left(j\right)}\right)\right)\right]$$

where the notations are:

- J(θ) is the cost function to be minimized;
- n is the number of training examples;
- $y^{\left(j\right)}$ is the actual label of the $j^{th}$ training example;
- $h_{\theta }\left(x^{\left(j\right)}\right)$ is the predicted probability that $\left(x^{\left(j\right)}\right)$ belongs to the positive class.

#### 3.5.4. Support Vector Machine

Supervised machine learning involves applying support vector machines (SVM) to address regression and classification problems. SVMs operate by identifying a hyperplane within an N-dimensional space, where N represents the number of features. The primary objective is to maximize the margin between data points associated with distinct classes. This approach facilitates the effective separation of classes in the feature space, making SVMs a versatile and powerful tool for supervised learning tasks. SVMs work very effectively in high-dimensional spaces and are ideal for situations with a noticeable margin of separation between classes.

In support vector machines, the objective function for classification tasks is to find the hyperplane that maximally separates the data into distinct classes. The main goal is to maximize the margin between the classes while minimizing the classification error. The formulation of the objective function depends on whether the problem is a linear or non-linear classification task. For a linearly separable case, the objective function aims to maximize the margin. The objective function for SVM is given in Equation (4).

(4) $$F\left(x\right)=\sin\left(w\cdot x+b\right)$$

where the notations are:

- w is the weight vector;
- x is the input feature vector;
- b is the bias term.

The margin is inversely proportional to the norm of the weight vector $\left\|w\right\|$. Therefore, the objective function to be maximized can be formulated as shown in Equations (5) and (6).

(5) $$Maximize=\frac{1}{2\;}{\left\|w\right\|}^{2}$$

subject to the constraint:

(6) $$F\left(x\right)=y_{j}\left(w_{j}\cdot x+b\right)$$

where the notation $w_{j}$ is the class label of the jth sample.

#### 3.5.5. Artificial Neural Network

Three primary layers comprise an artificial neural network (ANN): the input, the hidden, and the output layers. The data enters the system via the input layer, and the outputs emerge from the output layer. The backpropagation layer is an intermediate layer, which aims to change the weights to differ as little as possible from the target values. The ANN model that is being offered has an input layer that is made up of eight nodes, and then two hidden layers are made up of ten and eight nodes, respectively. The output layer comprises a single node because of the binary categorization nature of its output. Activation functions were applied to the first two layers using rectified linear units (ReLu), whereas the third layer was activated using sigmoid functions. In classification problems, regularization terms and a loss function are usually combined to form the objective function of an ANN. The goal function of a typical feedforward neural network used for classification is intended to be minimized during training. The cross-entropy loss, sometimes called log loss, is the most widely used loss function for classification tasks. The cross-entropy loss is frequently applied to binary classification tasks. The objective function is expressed in Equation (7).

(7) $$F\left(x\right)=-1/M\sum_{j=1}^{M}y_{j}\cdot \;log\left[{\hat{y}}_{j}\right]+\left(1-y_{j}\right)\cdot \log\left(1-{\hat{y}}_{j}\right)+\lambda R(\theta )$$

where the notation M is the number of training samples, $y_{j}$ is the true label of the jth sample, ${\hat{y}}_{j}$ is the predicted probability of the jth sample, θ represents the weights and biases of the parameters of the neural network, R(θ) is the regularization term, and λ is a regularization parameter that controls the strength of regularization.

## 4. Experimental Results and Performance Analysis

### 4.1. Performance Parameters

We have derived five key quality parameters in stroke prediction to evaluate model performance. Let us define the following terms based on stroke prediction. Now, we can express the evaluation metrics using these terms from Equations (8)–(12).

- True Positives (TP): the number of correctly predicted stroke cases;
- True Negatives (TN): the number of correctly predicted non-stroke cases;
- False Positives (FP): the number of incorrectly predicted stroke cases;
- False Negatives (FN): the number of incorrectly predicted non-stroke cases.

- Accuracy (*ACC*): accuracy measures the proportion of all correct predictions, the corresponding formula is shown in Equation (8).

(8) $$ACC=\frac{T_{p}+T_{n}}{T_{p}+T_{n}+F_{p}+F_{n}}$$

- Precision (*PR*): precision assesses the accuracy of positive predictions, the corresponding formula is shown in Equation (9).

(9) $$PR=\frac{T_{p}}{T_{p}+F_{p}\;}$$

- Recall (Sensitivity) (*RE*): recall, also known as sensitivity, evaluates the model’s ability to identify all positive instances, the corresponding formula is shown in Equation (10).

(10) $$RE=\frac{T_{p}}{T_{p}+F_{n}}$$

- Specificity (*SP*): specificity gauges the model’s capacity to correctly identify negative instances, the corresponding formula is shown in Equation (11).

(11) $$SP=\frac{T_{n}}{T_{n}+F_{p}}$$

- F1-Score (*F*1): The F1-score combines precision and recall into a single metric, the corresponding formula is shown in Equation (12).

(12) $$F1=2\times \frac{\left(PR\times RE\right)}{\left(PR+RE\right)}$$

- ROC Curve and AUC-ROC: the ROC curve graphs the true positive rate (recall) against the false positive rate (1—specificity) at different decision thresholds. The AUC-ROC quantifies the area under the ROC curve, indicating the model’s discriminatory power. These formulas provide quantitative ways to assess the performance of stroke prediction models based on their predictions of true positives, true negatives, false positives, and false negatives. Each metric serves a specific purpose and can help evaluate the model’s effectiveness in different aspects of stroke prediction.

### 4.2. Performance Results

Here, we summarize the suggested ML models’ prediction performance outcomes. In addition, how the risk factors affected the top-performing model’s classification performance.

**Figure 9:**
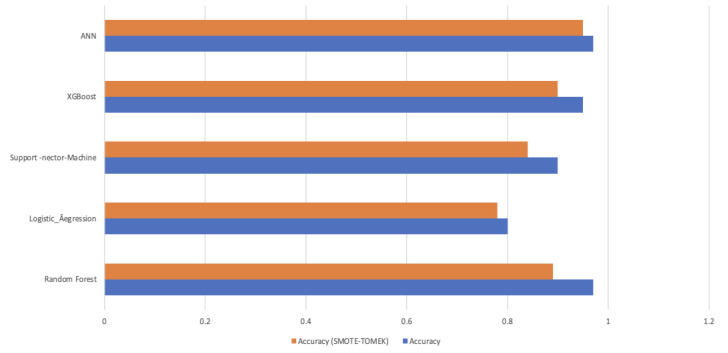
Mean of Prediction results with and without resampling.

In Table 4 and Figure 9, the “Actual” scenario (no resampling), all models have high accuracy. This suggests that the models are making very few positive predictions, and when they do, those predictions are accurate. However, they miss many positive cases. Under resampling techniques, like in Table 5 “Smote”, “Adasyn”, “Smote_Tomek”, and “Smote_Enn”, the models generally have lower accuracy and precision compared to the “Actual” scenario. However, their recall and F1-scores improved significantly. This indicates that resampling helped the models identify more positive cases, even though they may produce some false positives. “Undersampling” results in mixed performance. While it improves recall and F1-score for some models, it leads to lower performance for others, particularly in precision.

**Figure 10:**
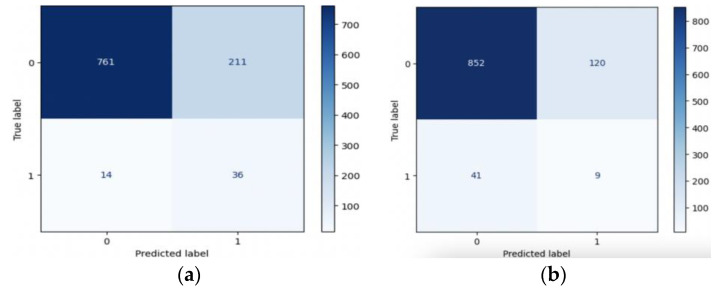

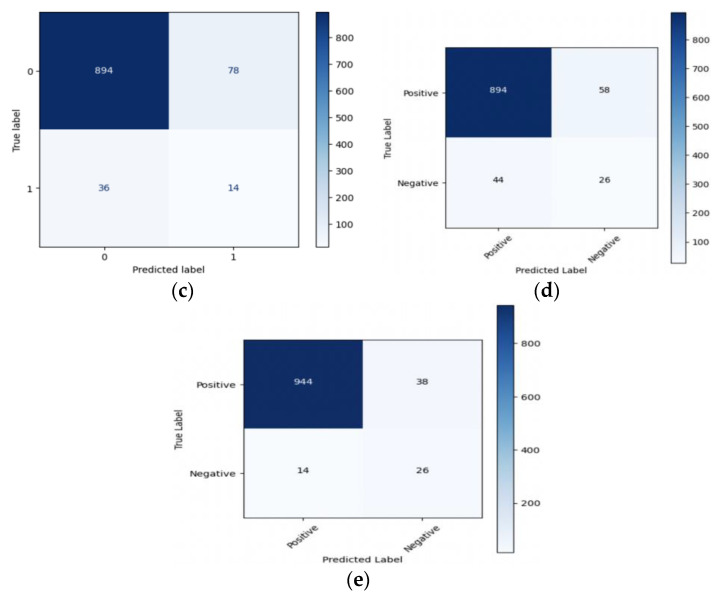
Confusion matrix of (**a**) LR, (**b**) SVM, (**c**) RF, (**d**) XGB, and (**e**) ANN.

**Figure 11:**
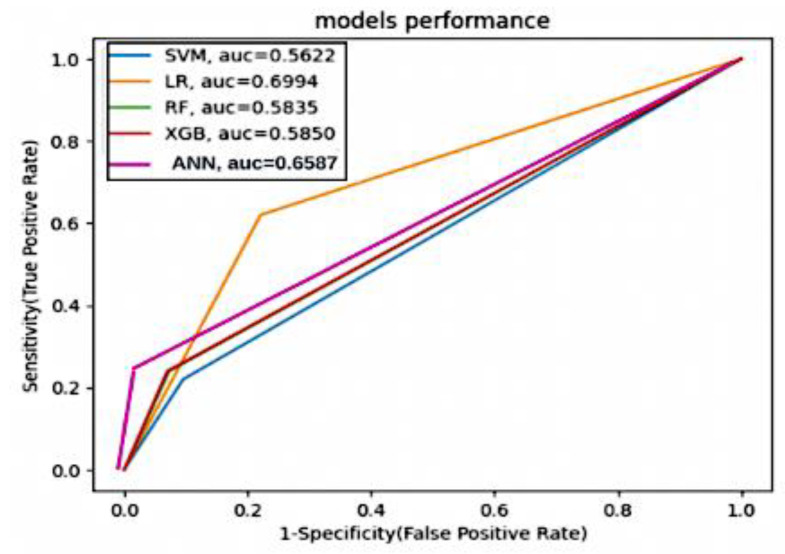
ROC_AUC curve of various machine learning models.

In summary, resampling techniques are applied to address class imbalance in the dataset. These techniques improve the models’ ability to correctly identify positive cases (stroke) while considering different trade-offs between precision and recall. The choice of resampling method should depend on the specific goals and requirements of the stroke prediction task, considering the importance of minimizing false negatives (missed stroke cases) and the tolerance for false positives (incorrectly predicted stroke cases). Figure 10 and Figure 11 present valuable insights into the performance of various machine learning models. Figure 10 displays the confusion matrix, while Figure 11 showcases the ROC_AUC curve for these models.

The ANOVA test is also used to determine whether there are significant differences in AUC values among the models. In this analysis, we used the AUC values for each model, and the ANOVA test results indicate significant differences in AUC values among the models based on the given *p*-value.

These hyperparameter configurations in Table 6 are tailored to optimize each algorithm’s performance based on the specific requirements for our stroke prediction dataset.

### 4.3. Model Interpretability

Prior studies on stroke prediction datasets have not elucidated the rationale behind model predictions. Our research, however, delves into the significance of each feature and clarifies the factors influencing specific model decisions. We offer both global and local perspectives. Locally, we pinpoint which features carry the most weight in individual test cases. This is achieved through the LIME explanation [32]. Globally, we assess feature contributions across a data set, such as all test data, using methods like permutation importance.

#### 4.3.1. Explainability Using LIME (Local)

**Figure 12:**
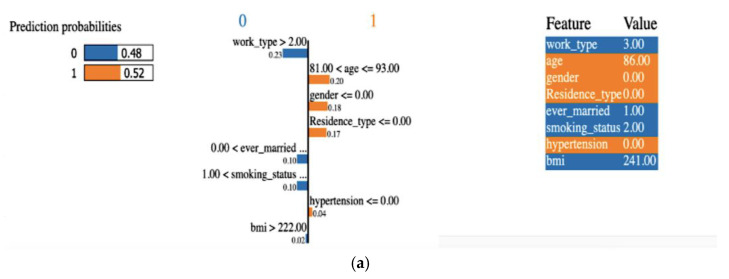

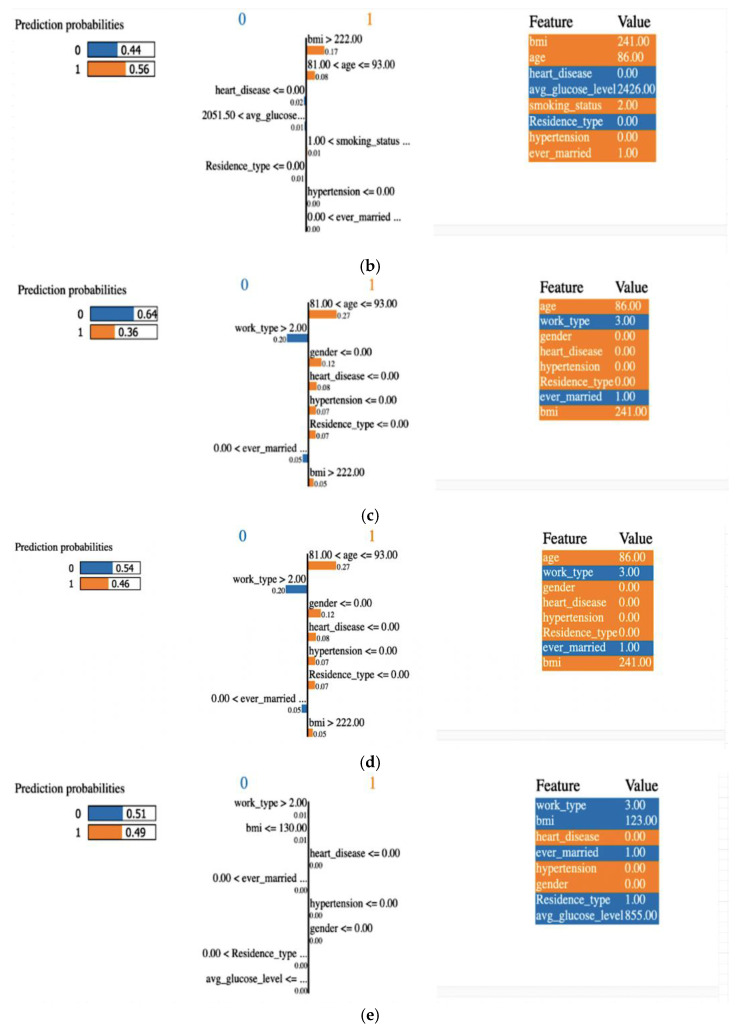
An explainer showing the LIME tabular explanations of the following: (**a**) LR, (**b**) SVM, (**c**) RF, (**d**) XGB, and (**e**) ANN.

Explainability of the outcome using LIME (local interpretable model-agnostic explanation) is a crucial aspect of ML model interpretability. LIME is a technique used to understand and provide insights into why an ML model makes specific predictions for individual data points or instances. A LIME tabular explainer was employed, as shown in Figure 12, for interpreting multiple ML models, including (a) LR, (b) SVM, (c) RF, (d) XGB, (e) ANN. This interpretability technique provided insights into the decision-making processes of these diverse models, offering a comprehensive understanding of their predictions and behaviors.

#### 4.3.2. Permutation Importance

An important feature in a predictive model is assessed using permutation importance in machine learning. It helps understand which features are the most important in making accurate predictions. Permutation importance is valuable for various purposes:

- Feature Selection: it helps identify the most relevant features in your dataset, allowing you to simplify and optimize your model;
- Model Evaluation: it provides insights into which features contribute the most to the model’s predictive power;
- Interpretability: permutation importance offers a way to explain model predictions by highlighting the importance of each feature.

**Figure 13:**
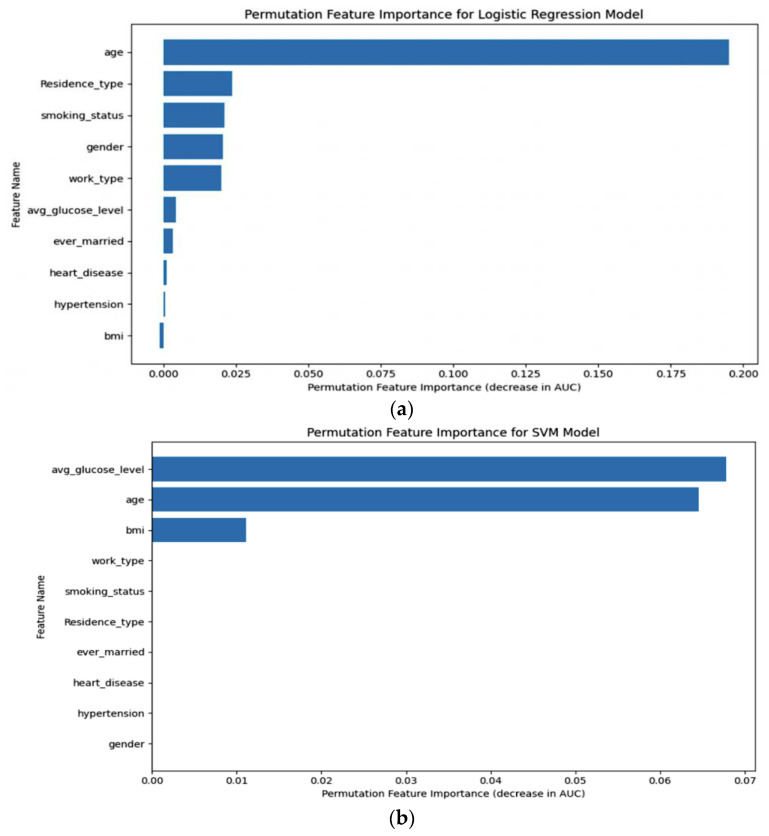

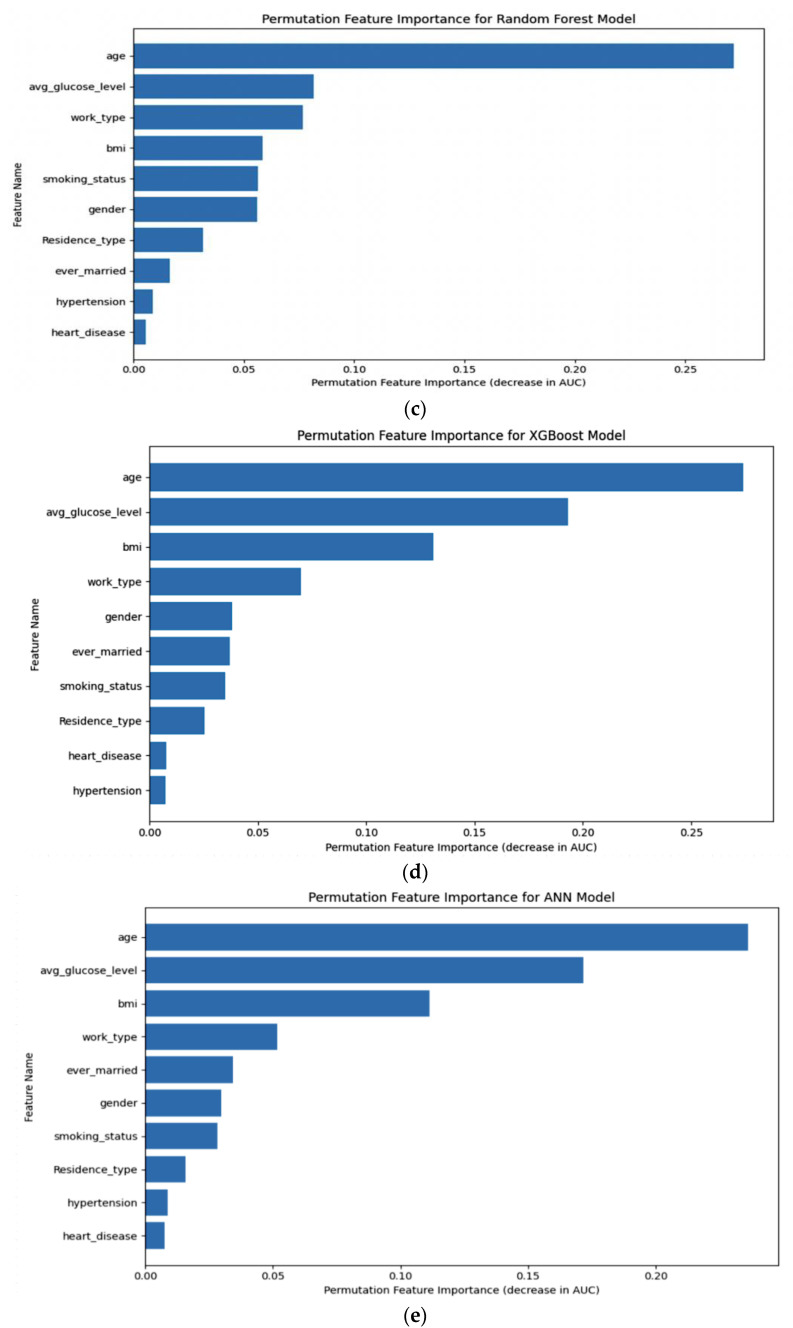
Permutation importance of (**a**) LR, (**b**) SVM, (**c**) RF, (**d**) XGB, (**e**) ANN.

Permutation importance was calculated, as shown in Figure 13, for five distinct machine learning models: (a) LR, (b) SVM, (c) RF, (d) XGB, and (e) ANN. This analysis allowed us to determine the significance of each model’s features by measuring how their random shuffling impacted the model’s performance, providing valuable insights into feature importance for predictive accuracy.

Healthcare systems could greatly benefit from using our suggested method, leading to better patient outcomes and enhancing current practices. In the real world, here are some concrete suggestions:

- Prevention initiatives and treatment program development;
- Coordinating with EHRs;
- Medical professionals’ decision-support tool;
- Prevention through patient education;
- Remote monitoring and telemedicine;
- Working in tandem with program that promote public health;
- Constantly enhancing models and feedback system.

## 5. Conclusions

Machine learning and deep learning models for detecting cardiac strokes are crucial. When it comes to detecting strokes early on, these models are vital for allowing prompt therapies and reducing the risk of long-term effects. The two main goals of our study are to improve the predictive accuracy and interpretability of basic neural networks and machine learning models used to forecast heart attacks.

Our all-inclusive model includes resampling methods, data leakage avoidance, and ANOVA feature selection. Making the model accessible to healthcare practitioners requires finding a compromise between model accuracy and interpretability. This study’s major addition is its multi-faceted approach to understanding the model’s inner workings and improving the accuracy and clarity of stroke prediction. The healthcare system may see less strain and better patient outcomes as a result of this in the long run.

Our current research provides a solid groundwork, but there are still opportunities for further investigation and improvement. To ensure our models are strong and can be applied to other populations, we should look into validating them externally on various datasets, and on continuously fine-tuning the model parameters to enhance prediction performance and investigate additional optimization strategies. These potential future directions highlight our dedication to expanding the area and improving our models for better results.

## Figures and Tables

**Table 1:** The Stroke Prediction Dataset includes the following attributes.

Attribute-Name	Attribute-Type	Attribute-Description
Id	Unique Identifier	A unique identifier for each patient.
Gender	Categorical	Gender of the patient is categorized as “Male”, “Female”, or “Other”.
Age	Numeric	Age of the patient.
Hypertension	Binary (0, 1)	In this case, a value of 1 denotes the presence of hypertension, whereas a value of 0 denotes its absence.
Heart_Disease	Binary (0, 1)	In this case, a value of 1 denotes the presence of a heart disease, whereas a value of 0 denotes its absence.
Ever_Married	Categorical	Patient’s marital status is coded as “No” or “Yes”.
Work_Type	Categorical	We can filter results by occupation or job status using terms like “children”, “Govt_jov”, “Never_worked”, “Private”, or “Self-employed”.
Residence_Type	Categorical	Classification of the patient’s place of residence, either “Rural” or “Urban”.
Avg_Glucose_Level	Numeric	A measurement of the average blood sugar level for the patient.
Bmi	Numeric	The patient’s body mass index.
Smoking_Status	Categorical	Patient’s smoking history; possible values are “formerly smoked”, “never smoked”, “smokes”, and “Unknown”.
Stroke	Binary (0, 1)	Whether the patient had a stroke (1) or not (0) is indicated by this value.

**Table 2:** Statistical Information about the Stroke Prediction Dataset.

Attribute Name	Age	Hypertension	Heart_Disease	Avg_Glucose_Level	Bmi	Stroke
Count	5110	5110	5110	5110	4909	5110
25%	25	0	0	77.245	23.5	0
50%	45	0	0	91.885	28.1	0
75%	61	0	0	114.09	33.1	0
MAX	82	1	1	271.74	97.6	1
MIN	0.08	0	0	55.12	10.3	0
Mean	43.226614	0.097456	0.054012	106.147677	28.893237	0.048728
STD	22.612647	0.296607	0.226063	45.28356	7.854067	0.21532

**Table 3:** Training datasets before and after different imbalance handling techniques using SMOTE.

SMOTE Techniques	Numbers in Class 0 (No Stroke)	Numbers in Class 1 (Stroke)
Before SMOTE	3771	156
SMOTE	3771	3771
ADASYN	3790	3771
SMOTE + TOMEK	3763	3763
SMOTE + ENN	2452	2033
SMOTE + Undersampling	2827	1131

**Table 4:** Mean of prediction results with and without resampling.

Model	Accuracy	Accuracy (SMOTE-TOMEK)
Random Forest	0.97	0.89
Logistic_Regression	0.80	0.78
SVM	0.90	0.84
XGBoost	0.95	0.90
ANN(Proposed)	0.97	0.95

**Table 5:** Evaluation metrics of various machine learning models under different sampling techniques.

Model	ACC	PR	RE	F1	Resample Technique Used
Random Forest	0.87	0.141414	0.264151	0.184211	Smote
Random Forest	0.89	0.128205	0.188679	0.152672	Adasyn
Random Forest	0.89	0.126761	0.169811	0.145161	Smote_Tomek
Random Forest	0.84	0.130435	0.339623	0.188482	Smote_Enn
Random Forest	0.90	0.152542	0.169811	0.160714	Undersampling
LR	0.77	0.128492	0.433962	0.198276	Smote
LR	0.78	0.133333	0.452830	0.206009	Adasyn
LR	0.78	0.135294	0.433962	0.206278	Smote_Tomek
LR	0.77	0.135593	0.603774	0.221453	Smote_Enn
LR	0.75	0.158537	0.490566	0.239631	Undersampling
SVM	0.83	0.140351	0.301887	0.191617	Smote
SVM	0.84	0.087719	0.188679	0.119760	Adasyn
SVM	0.84	0.109677	0.320755	0.163462	Smote_Tomek
SVM	0.79	0.140097	0.547170	0.223077	Smote_Enn
SVM	0.84	0.177966	0.396226	0.245614	Undersampling
XGB	0.89	0.127671	0.168911	0.145161	Smote
XGB	0.84	0.077819	0.184679	0.117760	Adasyn
XGB	0.90	0.155342	0.168911	0.107714	Smote_Tomek
XGB	0.89	0.127761	0.168911	0.146461	Smote_Enn
XGB	0.84	0.124535	0.339723	0.178482	Undersampling
ANN	0.94	1.000000	0.218868	0.137037	Smote
ANN	0.96	1.000000	0.18868	0.037037	Smote
ANN	0.95	1.000000	0.18868	0.037037	Smote_Tomek
ANN	0.93	0.210526	0.075472	0.111111	Smote_Enn
ANN	0.95	0.000000	0.000000	0.000000	Undersampling

**Table 6:** Hyperparameters used in the current study.

Algorithm	Parameter	Values
Random Forest	n_estimators	200
	max_depth	20
	min_samples_split	5
	min_samples_leaf	2
	max_features	‘sqrt’
	bootstrap	True
Logistic Regression	C	1
	penalty	‘l2’
	max_iter	200
SVM	C	1
	kernel	‘rbf’
	gamma	‘auto’
	degree	4
XGBoost	n_estimators	200
	max_depth	5
	learning_rate	0.1
	subsample	0.9
	min_child_weight	2
Artificial Neural Network	hidden_layer_sizes	(100)
	activation	‘relu’
	alpha	0.001
	learning_rate_init	0.01

## Data Availability

The authors utilized publicly available datasets.

## References

[1] Burns S.P., Fleming T.K., Webb S.S., Kam A.S.H., Fielder J.D., Kim G.J., Hu X., Hill M.T., Kringle E.A. (2022). Stroke recovery during the COVID-19 pandemic: A position paper on recommendations for rehabilitation. Arch. Phys. Med. Rehabil..

[2] Coute R.A., Nathanson B.H., Kurz M.C., Mader T.J., Jackson E.A. (2023). Disability-Adjusted Life-Years after Adult In-Hospital Cardiac Arrest in the United States. Am. J. Cardiol..

[3] Yang K., Chen M., Wang Y., Jiang G., Hou N., Wang L., Wen K., Li W. (2023). Development of a predictive risk stratification tool to identify the population over age 45 at risk for new-onset stroke within 7 years. Front. Aging Neurosci..

[4] Das M.C., Liza F.T., Pandit P.P., Tabassum F., Al Mamun M., Bhattacharjee S., Bin Kashem S. A comparative study of machine learning approaches for heart stroke prediction. Proceedings of the 2023 International Conference on Smart Applications, Communications and Networking (SmartNets).

[5] Emon M.U., Keya M.S., Meghla T.I., Rahman M., Al Mamun M.S., Kaiser M.S. Performance analysis of machine learning approaches in stroke prediction. Proceedings of the 2020 4th International Conference on Electronics, Communication and Aerospace Technology (ICECA).

[6] Ramesh G., Aravindarajan V., Logeshwaran J., Kiruthiga T., Vignesh S. (2022). Estimation analysis of paralysis effects for human nervous system by using Neuro fuzzy logic controller. NeuroQuantology.

[7] Caso V., Martins S., Mikulik R., Middleton S., Groppa S., Pandian J.D., Thang N.H., Danays T., van der Merwe J., Fischer T. (2023). Six years of the Angels Initiative: Aims, achievements, and future directions to improve stroke care worldwide. Int. J. Stroke.

[8] Ospel J.M., Kunz W.G., McDonough R.V., Goyal M., Uchida K., Sakai N., Yamagami H., Yoshimura S., RESCUE-Japan LIMIT Investigators (2023). Cost-effectiveness of endovascular treatment for acute stroke with large infarct: A United States perspective. Radiology.

[9] Singh M.S., Choudhary P., Thongam K. (2020). A Comparative Analysis for Various Stroke Prediction Techniques. Computer Vision and Image Processing.

[10] Pradeepa S., Manjula K.R., Vimal S., Khan M.S., Chilamkurti N., Luhach A.K. (2020). DRFS: Detecting Risk Factor of Stroke Disease from Social Media Using Machine Learning Techniques.

[11] Bandi V., Bhattacharyya D., Midhunchakkravarthy D. (2020). Prediction of Brain Stroke Severity Using Machine Learning. Int. Inf. Eng. Technol. Assoc..

[12] Nwosu C.S., Dev S., Bhardwaj P., Veeravalli B., John D. Predicting stroke from electronic health records. Proceedings of the 41st Annual International Conference of the IEEE Engineering in Medicine and Biology Society.

[13] Alotaibi F.S. (2019). Implementing Machine Learning Model to Predict Heart Failure Disease. Int. J. Adv. Comput. Sci. Appl. IJACSA.

[14] (2018). Ohoud Almadani, Riyad Alshammari: Prediction of Stroke using Data Mining Classification Techniques. Int. J. Adv. Comput. Sci. Appl. IJACSA.

[15] Kansadub T., Thammaboosadee S., Kiattisin S., Jalayondeja C. (2015). Stroke risk prediction model based on demographic data. Proceedings of the 8th Biomedical Engineering International Conference (BMEiCON).

[16] Khosla A., Cao Y., Lin C.C.Y., Chiu H.K., Hu J., Lee H. An Integrated Machine Learning Approach to Stroke Prediction. Proceedings of the 16th ACM SIGKDD International Conference on Knowledge Discovery and Data Mining.

[17] Shanthi D., Sahoo G., Saravanan N. (2009). Designing an artificial neural network model for predicting thrombo-embolic stroke. Int. J. Biom. Bioinform. IJBB.

[18] Sirisha U., Praveen S.P., Srinivasu P.N., Barsocchi P., Bhoi A.K. (2023). Statistical analysis of design aspects of various YOLO-based deep learning models for object detection. Int. J. Comput. Intell. Syst..

[19] Sirisha U., Chandana B.S. (2023). Privacy preserving image encryption with optimal deep transfer learning based accident severity classification model. Sensors.

[20] Stroke Prediction Dataset. https://www.kaggle.com/fedesoriano/stroke-prediction-dataset.

[21] Praveen S.P., Srinivasu P.N., Shafi J., Wozniak M., Ijaz M.F. (2022). ResNet-32 and FastAI for diagnoses of ductal carcinoma from 2D tissue slides. Sci. Rep..

[22] Srinivasu P.N., Shafi J., Krishna T.B., Sujatha C.N., Praveen S.P., Ijaz M.F. (2022). Using Recurrent Neural Networks for Predicting Type-2 Diabetes from Genomic and Tabular Data. Diagnostics.

[23] Zhao S., Guo Y., Sheng Q., Shyr Y. (2014). Advanced heat map and clustering analysis using heatmap3. BioMed Res. Int..

[24] Jonathan B., Putra P.H., Ruldeviyani Y. Observation imbalanced data text to predict users selling products on female daily with smote, tomek, and smote-tomek. Proceedings of the 2020 IEEE International Conference on Industry 4.0, Artificial Intelligence, and Communications Technology (IAICT).

[25] Rana C., Chitre N., Poyekar B., Bide P. Stroke prediction using Smote-Tomek and neural network. Proceedings of the 2021 12th International Conference on Computing Communication and Networking Technologies (ICCCNT).

[26] Goel G., Maguire L., Li Y., McLoone S. Evaluation of sampling methods for learning from imbalanced data. Proceedings of the Intelligent Computing Theories: 9th International Conference, ICIC 2013.

[27] Ye X., Xu W., Ye X., Long D., Yin Q., Huang B. (2023). Stroke Prediction Using the Trust Evaluation with Data Leakage Avoiding. Journal of Physics: Conference Series.

[28] Pathan M.S., Nag A., Pathan M.M., Dev S. (2022). Analyzing the impact of feature selection on the accuracy of heart disease prediction. Healthc. Anal..

[29] Awan S.E., Bennamoun M., Sohel F., Sanfilippo F.M., Chow B.J., Dwivedi G. (2019). Feature selection and transformation by machine learning reduce variable numbers and improve prediction for heart failure readmission or death. PLoS ONE.

[30] Clifford T., Bruce J., Obafemi-Ajayi T., Matta J. Comparative analysis of feature selection methods to identify biomarkers in a stroke-related dataset. Proceedings of the 2019 IEEE Conference on Computational Intelligence in Bioinformatics and Computational Biology (CIBCB).

[31] McHugh M.L. (2013). The chi-square test of independence. Biochem. Medica.

[32] An J., Zhang Y., Joe I. (2023). Specific-Input LIME Explanations for Tabular Data Based on Deep Learning Models. Appl. Sci..

